# Predictive Maintenance for Injection Molding Machines Enabled by Cognitive Analytics for Industry 4.0

**DOI:** 10.3389/frai.2020.578152

**Published:** 2020-11-16

**Authors:** Vaia Rousopoulou, Alexandros Nizamis, Thanasis Vafeiadis, Dimosthenis Ioannidis, Dimitrios Tzovaras

**Affiliations:** Centre for Research and Technology Hellas-Information Technologies Institute (CERTH/ITI), Thessaloniki, Greece

**Keywords:** cognitive analytics, artificial intelligence in manufacturing, predictive maintenance, ensemble learning, injection molding, Industry 4.0

## Abstract

The exploitation of big volumes of data in Industry 4.0 and the increasing development of cognitive systems strongly facilitate the realm of predictive maintenance for real-time decisions and early fault detection in manufacturing and production. Cognitive factories of Industry 4.0 aim to be flexible, adaptive, and reliable, in order to derive an efficient production scheme, handle unforeseen conditions, predict failures, and aid the decision makers. The nature of the data streams available in industrial sites and the lack of annotated reference data or expert labels create the challenge to design augmented and combined data analytics solutions. This paper introduces a cognitive analytics, self- and autonomous-learned system bearing predictive maintenance solutions for Industry 4.0. A complete methodology for real-time anomaly detection on industrial data and its application on injection molding machines are presented in this study. Ensemble prediction models are implemented on the top of supervised and unsupervised learners and build a compound prediction model of historical data utilizing different algorithms’ outputs to a common consensus. The generated models are deployed on a real-time monitoring system, detecting faults in real-time incoming data streams. The key strength of the proposed system is the cognitive mechanism which encompasses a real-time self-retraining functionality based on a novel double-oriented evaluation objective, a data-driven and a model-based one. The presented application aims to support maintenance activities from injection molding machines’ operators and demonstrate the advances that can be offered by exploiting artificial intelligence capabilities in Industry 4.0.

## 1 Introduction

Nowadays, the continuous accelerating pace of data creation and gathering from a wide range of sources such as sensors, posts to social media sites, transaction records, traffic data, pictures and videos, health data, mobile devices, and users’ activities led to significant changes in data analytics solutions by boosting machine learning (ML) and artificial intelligence (AI) methodologies to a wide range of domains (Salamanis et al., 2016; Vatrapu et al., 2016; Galetsi et al., 2020). The manufacturing domain was not an exception. The adoption of state-of-the-art algorithms and cutting-edge technologies in the years of Industry 4.0 enables the automation of processes and the creation of novel predictive maintenance solutions based on predictive and prescriptive analytics (Rojko, 2017).

Nonetheless, the full potential of the fast growing and changing data in manufacturing domain has not been unlocked yet. The application of human-like intelligence in the form of cognitive analytics in manufacturing domain is still in initial stages. Some initial approaches for cognitive manufacturing manage to improve analytics services’ quality and consistency. However, cognitive applications that can get smarter and more effective over time by learning from their interaction with data and by evaluating their own performance indicators in terms of precision, is still an ongoing activity. To this aim, the work presented in this study introduces a cognitive framework that exploits the capabilities of retraining mechanisms by continuous learning. Its application for predictive maintenance services in injection molding machines of a large electronics manufacturer’s shop floor demonstrates the advantages of this cognitive solution in terms of predictions’ accuracy.

An injection molding machine is commonly used in plastic processing industry and has to work continuously for long hours, so as to enable a continuous production line. A series of prediction, prevention, and inspection activities in order to alert machine problems and failures are vital for the normal and stable operation of a molding machine. This category of machines consists of different parts such as hydraulic, mechanical, and electrical parts that can cause failures. Usually, a failure is related to abnormal rise of temperature in an injection molding machine. The problem can be related to various factors such as problems in cooling system, improper pressure regulator, and high pressure in hydraulic system alongside with long period of overheating. Besides the temperature, the abnormal generated noise can be a real-time failure indicator if this kind of data is available. Damaged hydraulic and mechanical components can lead to significant variations of sound. The detection of substandard products with lower quality could be the last indicator of an injection molding machine failure.

The current study introduces a predictive solution based on the application of cognitive analytics in feature parameters coming in real time from injection molding machines by using IDS connectors (Otto et al., 2019; Otto and Jarke, 2019). The proposed predictive models aim to detect abnormalities out of the available temperature, pressure, and energy consumption data. Since both labeled and unlabeled data exist in the aforementioned machines, supervised and unsupervised learning algorithms have been deployed based on the availability or not of ground truth in the data, respectively. Ensemble learning was implemented upon different learners in order to combine their independent decisions and boost the fault detection mechanism. In the case that ground truth is available by the machines, the Adaptive Boosting (Freund and Schapire, 1995; Nath and Behara, 2003; Schapire and Freund, 2012) ensemble technique was applied to the deployed supervised learning methodologies in order to increase predictive performance. Accordingly, the major voting method is implemented on the top of unsupervised learning. As the injection molding machine condition monitoring forms a nonstationary environment, an adaptive and evolving approach is presented, capable of accommodating changes. So, the produced predictive models are continuously evaluated through a double-oriented evaluation objective, a data-driven and a model-based one. The latter enables a novel real-time self-retraining functionality for boosting the cognitive capabilities of the proposed solution.

The paper is structured as follows. Following the Introduction, a related work review is presented. Section 3 contains a detailed description of the proposed methodology, while Section 4 demonstrates the experimental results of the study. Finally, the conclusions of the study are drawn at Section 5.

## 2 Related Work

There are several available methodologies, concepts, and solutions related to predictive maintenance services in Industry 4.0. The selected related work in this section is presented by the perspectives of cognition in manufacturing domain, predictive maintenance for injection molding machines, and ensemble methods for the enhancement of predictive services. The three aforementioned categories constitute the main advances of the current work and the corresponding bibliography was considered as the most suitable one to be mentioned in this section.

The advances in nowadays software and hardware technologies enable computer systems to mimic human brain activities and acquire cognition capabilities. The alleged capabilities introduce cognitive computing which is based on software that learns by itself, without reprogramming, and it is able to automate cognitive tasks. Industry 4.0 solutions have adopted various cognitive computing approaches for predictive maintenance, planning optimization, and performance and quality improvement. To this direction, the concept of the Cognitive Factory is supposed to be flexible, adaptable, reliable, and efficient in various momentary situations (Zaeh et al., 2009). This type of factory is moving from perception to action by using continuous learning and cognitive mechanisms. The advantages, disadvantages, and future challenges in the field of cognitive manufacturing have been widely studied (Bannat et al., 2011; Iarovyi et al., 2015). Iarovyi et al. (2015) present a documentation of different architectures for cognitive manufacturing systems that can be benefited from Industrial Internet of Things and cognitive control. Bannat et al. (2011) investigate methods to realize cognitive control and cognitive operation of production systems by highlighting self-optimizing and self-learning procedures. Iarovyi et al. (2015) again propose an architecture for cognitive manufacturing systems by combining approaches from PLANTCockpit (2012) and CogNetCon (Boza et al., 2011), enabling efficient data integration in manufacturing environments and providing connectivity between data on shop floor level and data in MES, ERP, and other systems. A cognition layer in the architecture contains a cognition engine, a model repository, and knowledge representation components. By adopting the aforementioned components, the architecture targets higher-level decision-making, self-learning, reconfiguration, and self-optimization in manufacturing domain.

Comprehensive research has been held toward predictive maintenance in manufacturing, including the study and analysis of sensor data and industrial machines for early fault detection, condition base monitoring, and decision support systems. Specifically, injection molding machines have been investigated as a real-world industrial application of predictive analytics (Gatica et al., 2016; Park et al., 2016; Jankov et al., 2017). An overview of industrial analytics methods and applications for predictive maintenance in manufacturing is presented by Gatica et al. (2016), encompassing an injection molding machines’ use case. The work of Gatica et al. (2016) classifies the machinery analytic approaches in offline and online analysis. The offline analytics contain the “hypothesis-driven” strategy which is based on the analysis of the machine behavior and the “data-driven” strategy which focuses on exploration of the information provided by sensors and machine logs. Online analytics resolve predictive maintenance through data monitoring and machine state recognition by employing machine learning models. Jankov et al. (2017) introduce another real-time anomaly detection system dealing with injection molding machines. The presented system performs anomaly detection using K-means for cluster finding and Markov model for data training. Jankov et al. (2017) describe a custom-built system and concentrate on the system’s performance through parallel and real-time processes. Furthermore, the method proposed by Park et al. (2016) distinguishes the different maintenance items of an injection molding and maps each one of these items to selected parameters in the collected data. Thereafter, a live parameters’ monitoring process takes place and abnormal trends or patterns are detected based on statistical techniques. The detected abnormalities for different machine’s parts are available to the maintenance operators. Last but not least, in the field of predictive analytics in manufacturing, a study which introduces an application on industrial ovens is worth mentioning (Rousopoulou et al., 2019). This study’s methodology could as well be applied to injection molding machines, as it concerns the usage of both existing machine sensors with their log data and deployed sensors and achieves early fault diagnosis in an industrial machine.

Finally, on the subject of ensemble learning, ensemble techniques contribute to the performance of supervised and unsupervised machine learning models and enhance the predictive maintenance analytics solutions. A recent work regarding ensemble learning proves the improvement of individual learning models in terms of accuracy as well as training time by implementing ensemble learning and creating an integrated model through majority voting, experimenting on refrigerator system’s datasets (Zhang et al., 2020). Additionally, in terms of assessing the ensemble techniques, a thorough benchmarking evaluation of outlier detection algorithms was reviewed (Domingues et al., 2018). Unsupervised machine learning algorithms were tested and compared on multiple datasets, highlighting their strengths and weaknesses. Within this context, an application of unsupervised outlier detection on streaming data containing travel booking information was implemented (Domingues et al., 2016). The study of Domingues et al. (2016) performs fraud detection by examining aggregation functions and interpolation in order to address unsupervised ensemble learning.

## 3 Methodology

The proposed methodology constructs a real-time anomaly detection solution with cognitive retraining, applied to an industrial machine. Starting with the training of historical data by state-of-the-art ML algorithms and meta-learners, prediction models are created. The models are fed with live incoming data streams and detect abnormalities in real time. This live monitoring process is enhanced by an automated retraining mechanism which inspects the characteristics of the new input data and the models’ performance in order to update the prediction models and maintain the high performance of the fault detection system. The methodology consists of the following components:

1. Data Preparation
2. Online Training
3. Ensemble Learning
4. Live Prediction
5. Cognitive Check

**FIGURE 1:**
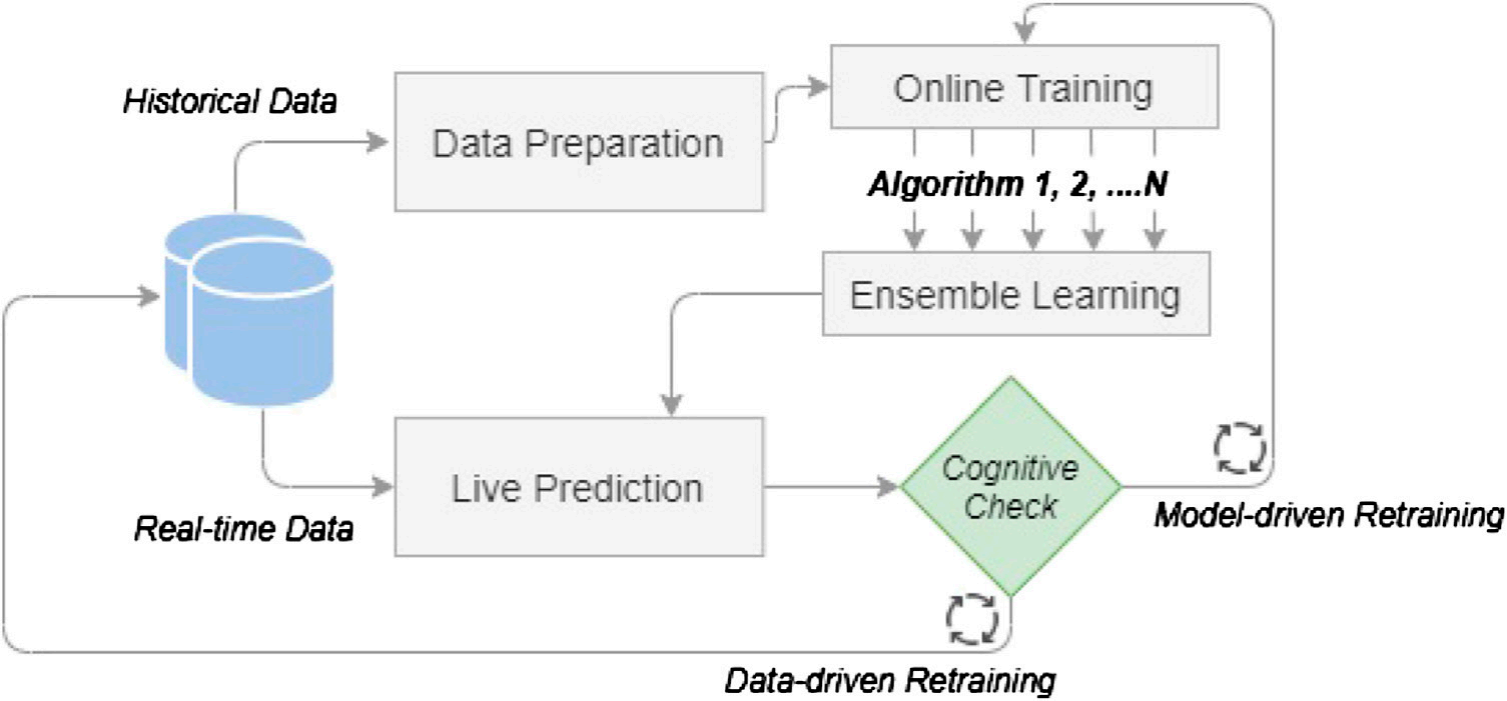
Overview of the cognitive system’s methodology architecture.

Figure 1 is an illustration of the proposed methodology. The process starts with historical data that are inserted for data preparation. The online training includes the training of several algorithms which are afterward enhanced by the ensemble learning step. The above steps examine and determine the optimum models for live prediction which performs real-time anomaly detection. New live data are coming through the predictive models which are constantly evaluated by a cognitive check and updated by automatic model retraining. The proposed method aims to form a regularly updated system which can monitor an injection molding machine and predict machine or part failures in order to reduce or even prevent machine downtime and save time and cost in the production line. The remainder of this chapter is a detailed description of the proposed solution, underlining the methods and algorithms combined in a complete anomaly detection pipeline.

### 3.1 Data Preparation

The current study’s dataset is composed of measurements from injection molding machines which carry out the process of shaping rubber or plastic parts by injecting heated material into a mold. Specifically, the injection molding process deals with the fabrication of plastic components for electric shavers. The available measurements are expressed in time-series format, including different kinds of measurements, such as temperature, pressure, energy consumption, and time. Anomalies in time-series data indicate “bad” shots during injection, which leads to rejected products.

Six different injection molding machines are available in the dataset; four of them contain labeled data and the other two contain unlabeled data. Table 1 shows the features that each machine contains. The labeled datasets include a quality indicator feature (label). The zero label suggests a normal instance, while a nonzero label indicates an abnormality in machine’s performance. The values of the quality indicator feature correspond to a specific error, but since this aspect goes beyond the context of the current research, the label is converted to binary, with zero meaning normal and one meaning abnormal instance.

**TABLE 1:** The available datasets from injection molding machines.

Feature description	Injection molding machine #
Timestamp	1, 2, 3, 4, 5, 6
Part counter	1, 2
Bad part counter indicator	1, 2
Last value of cycle time	1, 2, 3, 4, 5, 6
Peak of hold pressure	1, 2, 3, 4, 5, 6
Injection pressure	1, 2
Peak of injection pressure	1, 2, 5, 6
Flow number	1, 2, 3, 4, 5, 6
Melt cushion	1, 2, 3, 4, 5, 6
Hydraulic pressure	1, 2
Peak of injection pressure	1, 2, 3, 4, 5, 6
Value of switchover position	1, 2
Corrected position of plasticizing	1, 2, 3, 4, 5, 6
Clamp force	1, 2, 3, 4, 5, 6
Value of mold protection time	1, 2, 5, 6
Oil temperature	1, 2, 3, 4
Temperature of zone X	1, 2, 3, 4, 5, 6
Number of cavities	3, 4
Cooling time	3, 4, 5, 6
Screw position	3, 4, 5, 6
Injection time	3
Switchover pressure	3, 4, 5, 6
Shot counter	3, 4
Bad shot counter	3, 4
Peak of back pressure	3, 4, 5, 6
Plasticizing time	3, 4, 5, 6
Set value of temperature of zone X	3, 4, 5, 6
Heating energy consumption	3, 4, 5, 6
Motor energy consumption	3, 4, 5, 6
Total energy consumption	3, 4, 5, 6
Quality indicator (label)	3, 4, 5, 6

In order to transform raw data into refined information assets, first cleansing of the data takes place. The constant, empty, and duplicated columns are removed from the dataset. The columns with insignificant variance measure, namely, lower than 0.01, are removed as well. In order to resolve the real-world data inconsistency or incompleteness, the following preprocessing and cleansing methods are implemented for the data preparation step:

- Interpolation for estimating missing values between known data points is used.
- Zero variables are eliminated by zero removal process, as well.
- Normalization is used in order to scale and translate each feature individually in the range between zero and one.

After the preprocessing phase the features of each dataset are reduced as shown in Table 2. The dataset is split into 70% of samples for training and 30% for testing.

**TABLE 2:** The available datasets from injection molding machines.

Dataset	Label	Initial feature size	Final feature size
Injection molding machine 1	Yes	63	48
Injection molding machine 2	Yes	63	47
Injection molding machine 3	Yes	75	42
Injection molding machine 4	Yes	73	47
Injection molding machine 5	No	34	23
Injection molding machine 6	No	34	21

### 3.2 Online Training

A set of supervised and unsupervised learners is applied to the machine data, depending on the needs of each injection molding machine. The nature of the data led us to address the anomaly detection problem through classification and clustering methods. The algorithms reported at this chapter were selected for the current research due to the sufficient results that they have achieved in terms of prediction models’ performance. However, the solution is extensible enough, so as to incorporate new methods within the overall architecture.

The online training of labeled datasets is addressed by well-known supervised training methods. The methods classify the input datasets and are capable of creating prediction models that detect faults in this input. Specifically, Support Vector Machines (SVMs) classifier is one of the most convenient and widespread classification algorithms, able to construct a hyperplane as a decision boundary as the maximum margin between classified classes based on kernel functions. In this work, two kernel functions are applied: Polynomial and Radial Basis Function. Decision tree learning is a technique for approximating discrete-valued functions, in which the learned function is represented by a decision tree (or classification tree or learning tree) (Lee and Siau, 2001). Random forest is an ensemble of decision trees and each decision tree is constructed by using a random subset of the training data, while the output class is the mode of the classes decided by each decision tree (Breiman, 1999). Finally, Artificial Neural Networks (ANNs) are used and especially Back Propagation Network (BPN) which is a feed-forward model with supervised learning (Rumelhart et al., 1986), and for the need of this work a fully connected neural network is used with one hidden layer.

On the other side, for the online training of unlabeled datasets, unsupervised learning techniques were implemented aiming to detect anomalies through data clustering. Thus, in this section we present state-of-the-art unsupervised learning methodologies that have been used in this work. DBSCAN is the data clustering algorithm which discovers clusters of arbitrary shape in spatial dataspaces with noise. Next is the Local Outlier Factor (LOF), which provides a factor of how close a data point is to its neighbors in respect to its neighbor being also close to it. The One-Class Support Vector Machine (One-Class SVM) algorithm classifies the points that lie outside some boundaries of the data space as outliers. Finally, K-means iteratively tries to partition the dataset into clusters with each data point belonging to only one cluster.

### 3.3 Ensemble Learning

On the top of individual learners, ensemble methods are techniques that utilize multiple models so as to combine them in order to produce improved results. Ensemble methods are incorporated into our methodology so as to generate a more accurate solution comparing with the results of single models. Our methodology proposes two ensemble algorithms for supervised and unsupervised learners, Adaptive Boosting and majority voting, respectively.

Adaptive Boosting (or AdaBoost) technique is a conjunction of many classification algorithms (also called weak learners), either from different families or from the same family with different internal parameters, aiming to improve classification performance compared to a single and simple classification algorithm. AdaBoost takes as input the outcome of a weak learner and iteratively improve it by recalculating its weights for the incorrectly classified cases in the training set. Adaboost is adaptive in the sense that subsequent weak learners are tweaked in favor of those instances misclassified by previous classifiers. There are many forms of boosting algorithms (Nath and Behara, 2003; Schapire and Freund, 2012), but the most popular is the one where the weak classifiers are decision trees (Freund and Schapire, 1995). In this work, we use the AdaBoost SAMME–Stagewise Additive Modeling using multiclass exponential loss function, which is an extension of AdaBoost.M1 algorithm, so as to perform both two-class and multiclass classification scenarios.

Majority vote (Jung and Lease, 2012) is a simple method for generating consensus among different algorithms by picking the label receiving the most votes. The rationale of the method is to calculate the average label coming from multiple learners and round according to a decision threshold. The majority vote is used as an enhancement for the individual learner’s anomaly detection. It is also used as a replacement for the ground truth in case of unlabeled datasets, especially on the cognitive check taking place in the live prediction step of the proposed methodology (Section 3.5).

The aforementioned ensemble methods are applied automatically to the trained algorithms. In the case of supervised learners the AdaBoost methods provide an enhancement in terms of accuracy of single learners. In the case of unsupervised learners, the majority voting is used as a combined learner which performs better than a single learner or as a substitute of ground truth values in order to facilitate the cognitive check described in the next sections.

### 3.4 Live Prediction

When the training phase is over each machine dataset acquires one prediction model and its metadata (preprocessing models, statistic measures, and logs). The prediction model with the highest accuracy metric prevails in case of supervised learning, whereas the model with the highest silhouette score prevails in case of unsupervised models. Both supervised and unsupervised optimal models are specified as the “default” model. These models perform the outlier prediction on new live incoming data streams. The machine data are constantly monitored and for each instance of measurements, the system recognizes normal behavior or detects anomalies.

The incoming data stream is being edited and brought to the same format as the training dataset. The preprocessing methods used in training phase are applied precisely to the input data stream which will next be imported in the “default” prediction model. The anomaly detection results are kept in order to be used for evaluation and cognitive updating. The live monitoring procedure is constantly operating and updated in the aforementioned way.

**FIGURE 2:**
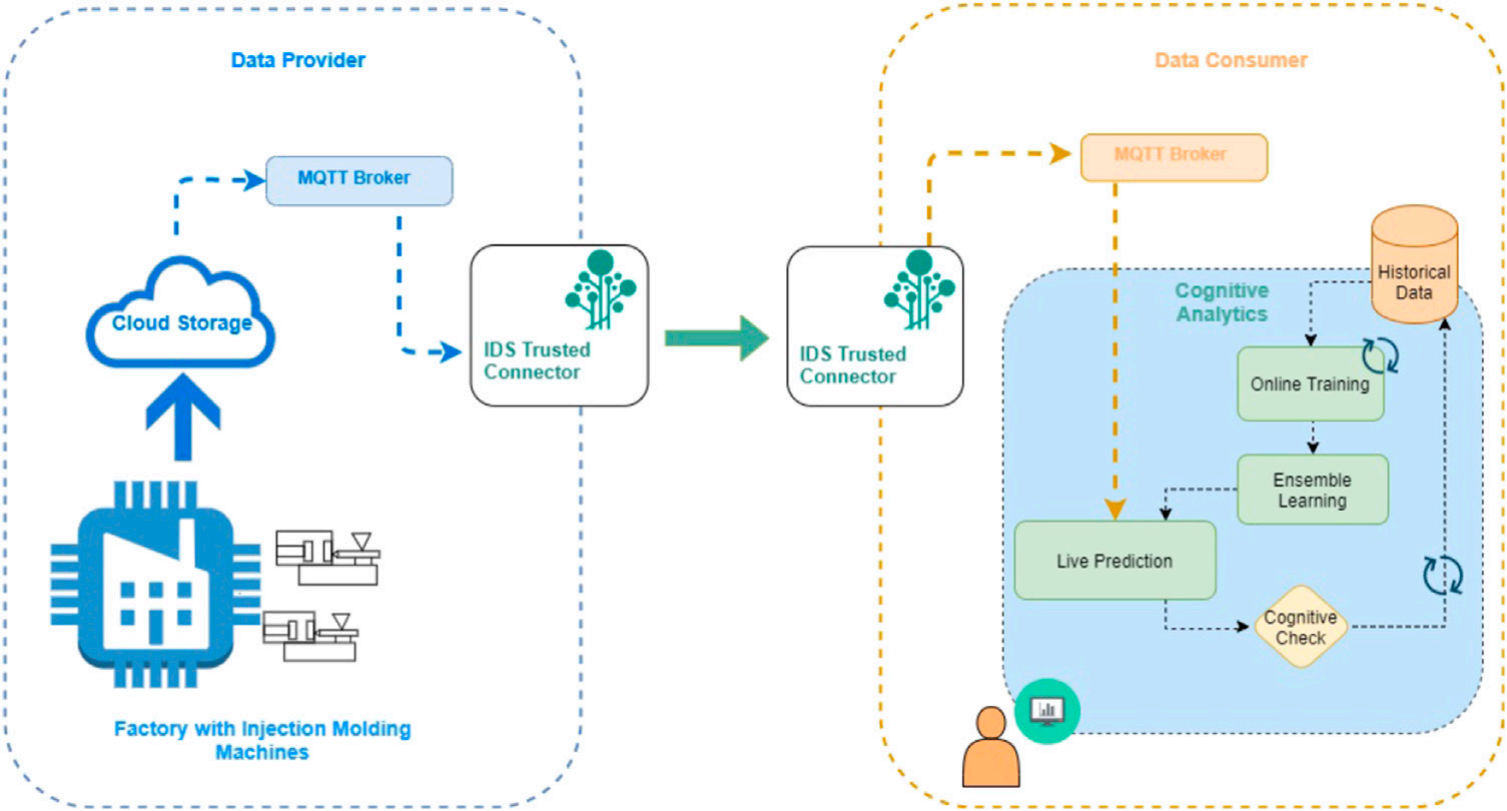
Overview of the connection of the injection molding machines factory site with the proposed cognitive analytics.

Technically, the injection molding machine live data are retrieved through a custom IDS connector system that was set up for the purposes of the presented work. Figure 2 illustrates the integration of real-time machine data with the cognitive analytics. The system is based on two IDS Trusted Connectors. The first connector is deployed on the factory site. The machines send data to the cloud infrastructure that is available to the factory and from there, the data are provided to IDS connector through an MQTT Broker. The factory cloud repository is the data provider of IDS architecture, whereas the cognitive analytics framework is the data consumer. A second IDS Trusted Connector was set up on consumer site alongside with a MQTT Broker in order to enable data exchange with the data provider. The IDS Trusted Connectors were selected because they offer an open platform which connects sensors with cloud infrastructures and other connectors in a secure and trusted way. In particular, the connectors are based on containers logic and provide apps isolation. They are isolated from each other and from the Internet. Furthermore, the connectors offer cross-enterprise authorization based on identity tokens. Another advantage of the data exchange between connectors is the ability to control and document the data usage. In addition to access control, the usage control allows for controlling data flows between apps and connectors. Based on the aforementioned advantages of IDS Trusted Connectors, they were selected as the ideal candidates to support the major requirement for secure transmission of the sensitive and private industrial data.

### 3.5 Cognitive Check

A cognitive mechanism is implemented at this point toward automated update of the prediction models. This mechanism triggers the retraining of the running models in two specific circumstances:

- the dataset’s characteristics are changed
- the model’s performance starts to downgrade

The new data that are constantly inserted in the prediction models are reassessed in order to capture possible variations compared to the historical data. The variance of the features of the historical datasets is stored and every new incoming data stream is compared with this value. If the new measurements are not statistically related to the training dataset, then the model training has to be repeated on the new dataset. In any case, the cognitive mechanism observes repeated measurements with variations until it finally triggers the retraining of the dataset, so as to eliminate accidental discrepancies of the machine live data.

The retraining is also activated by monitoring of the prediction model performance. An Initial Prediction Window (IPW) is determined at the training phase, which is a specific number of real-time predictions tested against the real ones when those are available. In case of the machines with labeled data, the real values are given and compared to the predicted ones. In case of the machines with unlabeled data, the result of major voting method substitutes the labels of data instances and is compared with the predicted results in order to extract the performance metrics. In both cases, a confusion matrix is created and the metrics, precision, recall, accuracy, and f-measure are calculated.

Based on the values of f-measure, the IPW is changed (increases or decreases) or remains the same. More specifically, a minimum and maximum value are defined for the IPW values, along with a threshold for the f-measure value. Starting from the maximum IPW value, f-measure is calculated for this window. If f-measure exceeds the defined threshold, the training model remains as it is, whereas IPW increases by 10 if f-measure is higher than 90%, decreases by 10 if f-measure is lower than 80%, and remains as it is if f-measure falls between 80 and 90%. This process is repeated until the IPW equals the minimum IPW. In case that f-measure falls behind the defined threshold, the retraining mode is triggered and the IPW value resets to the maximum value.

## 4 Experimental Results and Comparison With Prior Work

The methodology described in the previous chapter refers to a dynamic and automatic system for real-time anomaly monitoring. The online training functionality is the basis for the live monitoring and is automatically triggered according to the cognitive mechanism. Since it is a live system, in order to evaluate its functionalities and performance, an indicative instance of training and testing of the prediction models is presented below. Furthermore, an experiment of the cognitive mechanism is presented at this chapter, showing the robustness of the proposed method. Finally, a comparison between our method and prior related work is performed.

### 4.1 Evaluation Metrics Overview

In order to assess our supervised models, we use the measures of precision, recall, accuracy, and f-measure, which are computed from the contents of the confusion matrix of the classification predictions. Because of the fact that we do not have binary classification, all the evaluation metrics are computed accordingly. From the confusion matrix true positive and false positive cases are denoted as TP and FP, while true negative and false negative are denoted as TN and FN, respectively. Precision is the ratio of predicted true positive cases to the sum of true positives and false positives and is given by the equation

$$\text{Precision}=\frac{\text{TP}}{\text{TP}+\text{FP}}.$$

Recall is the proportion of the true positive cases to the sum of true positives and false negatives and is given by the equation

$$\text{Recall}=\frac{\text{TP}}{\text{TP}+\text{FN}}.$$

Accuracy is the fraction of the total number of predictions that were correct and is given by the equation

$$\text{Accuracy}=\frac{\text{TP}+\text{TN}}{\text{TP}+\text{FP}+\text{TN}+\text{FN}}.$$

Precision or recall alone cannot describe a classifier’s efficiency. Therefore, f-measure is introduced as a combination of these two metrics. It is defined as twice the harmonic mean of precision and recall and is the metric we will be most referring to. The equation of f-measure is given below:

$$\text{f}-\text{measure}=\frac{2\times \text{Precision}\times \text{Recall}}{\text{Precision}+\text{Recall}}.$$

A value closer to one means better combined precision and recall of the classifier, whereas lower values imply worst accuracy or precision or both.

Accordingly, the unsupervised model assessment is performed by four clustering performance evaluation metrics: Silhouette Coefficient, Calinski–Harabasz index, Davies–Bouldin index, and Dunn index. Those are metrics for evaluating clustering algorithms following an internal evaluation scheme, where the metric result is based on the clustered data itself. The Silhouette Coefficient is an example of evaluation using the model itself (Rousseeuw, 1987). The Silhouette Coefficient for a single sample is given as

$$\text{Silhouette}=\frac{b-a}{\text{max}\left(a,b\right)},$$

where *a* is the mean distance between a sample and all other points in the same class and *b* is the mean distance between a sample and all other points in the next nearest cluster. The Silhouette Coefficient for a set of samples is given as the mean of the Silhouette Coefficient for each sample. Higher Silhouette Coefficient scores indicate a model with better defined clusters.

Another evaluation metric, in case that the ground truth labels are not known, is the Davies–Bouldin index (Davies and Bouldin, 1979). The “similarity” between clusters is measured by this metric by comparing the distance between clusters with the size of the clusters themselves. The Davies–Bouldin index is specified as

$$\text{DB}=\frac{1}{K}\overset{k}{\underset{i=1}{\sum }}{\text{max}}_{i\neq j}R_{ij},$$

where $R_{ij}$ is the similarity measure defined as

$$R_{ij}=\frac{s_{i}+s_{j}}{d_{ij}}$$

for each cluster $C_{i}$ for *i* = 1, …, *k* and its most similar one $C_{j}$:

- $s_{i}$ is the average distance between each point of cluster *i* and the centroid of that cluster.
- $d_{ij}$ is the distance between the cluster centroids *i* and *j.*

The lowest possible score is zero and values closer to zero suggest a better partition. Next is the Calinski–Harabasz index also known as the Variance Ratio Criterion (Caliński and Harabasz, 1974). The index is the ratio of the sum of the between-clusters dispersion and inter-cluster dispersion for all of them:

$$\text{CH}=\frac{\text{tr}\left(B_{k}\right)}{\text{tr}\left(W_{k}\right)}\times \frac{n_{E}-k}{k-1},$$

where $\text{tr}\left(B_{k}\right)$ is trace of the between-group dispersion matrix and $\text{tr}\left(W_{k}\right)$ is the trace of the within-cluster dispersion matrix defined by

$$W_{k}=\sum_{q=1}^{k}\sum_{x\in C_{q}}\left(x-c_{q}\right){\left(x-x_{q}\right)}^{T}$$

and

$$B_{k}=\sum_{q=1}^{k}h_{q}\left(c_{q}-c_{E}\right){\left(c_{q}-c_{E}\right)}^{T},$$

where $C_{q}$ is the set of points in cluster *q*, $c_{q}$ is the center of cluster *q*, $c_{E}$ is the center of *E*, and $n_{q}$ is the number of points in cluster *q*. The higher Calinski–Harabasz score implies a model with better defined clusters. Last is Dunn index (Dunn, 2008), another metric that aims to identify the compact sets of clusters and the well-separated ones. The metric is given by the following equation:

$$\text{DI}=\frac{\underset{1\leq i\leq j\leq m}{\text{min}}\delta \left(C_{i},C_{j}\right)}{\underset{1\leq k\leq m}{\text{max}}{\text{Δ}}_{k}},$$

where $\delta \left(C_{i},C_{j}\right)$ is the distance between clusters $C_{i}$ and $C_{j}$, and ${\text{Δ}}_{k}$ is the intracluster distance within cluster $C_{k}$. The higher the Dunn index value is, the better the model performance is.

### 4.2 Training Simulation Results

The section of experimental results regarding online training is divided into three subsections. In the first subsection, the performance of all tested classifiers is presented, while in the second subsection the boosted version of the classifier with the best predictive performance among tested ones is presented. The third subsection presents results from the unsupervised training. Out of all experiments conducted in this research in order to test and evaluate the proposed methodology, some indicative results are given below in order to show the potential of the system and the attempt to create a compound solution for the injection molding machines. The evaluation of the system presented at this point focuses on both the functionalities of the proposed solution and the performance of the available algorithms presented in Section 3.

#### 4.2.1 Nonboosted Version of Classifiers

In order to evaluate the predictive performance of tested classifiers, a series of 100 Monte-Carlo simulations was performed, for each parameter schema. The idea behind Monte-Carlo simulations is the generation of a large number of synthetic datasets that are similar to experimental data. In the case of time series the simulation setup of the match for Monte-Carlo realizations is 100-fold cross-validation. For SVM-POLY, parameter θ takes the values θ *=* (start = 30, end = 60, step = 6) and the polynomial degree *p* takes the values *p* = (2, 5, 1). For SVM-RBF, parameter σ varies the same as θ and the constant *C* as C = (1,000, 7,000, 2,000). In Tables 3, 4, we present the simulation results of SVM-POLY and SVM-RBF classifiers, percentage averages for 100 Monte-Carlo iterations for precision, recall, accuracy, and f-measure. The classic BPN has a single hidden layer and the number of neurons varies as *n* = (100, 200, 20). The simple decision tree was tested as is while the random forest has an ensemble of estimators = (20, 100, 20) decision trees. In Tables 5, 6, we present the simulation results of BPN and random forest classifiers, percentage averages for 100 Monte-Carlo iterations for precision, recall, accuracy, and f-measure. From all the simulation results presented in Tables 3-6, it is more than clear that random forest classifier outperforms SVM-POLY, SVM-RBF, and BPN for about 11–12%. Thus, random forest is the one classifier that will be promoted to test also in its boosted form.

**TABLE 3:** Averages of precision, recall, accuracy, and f-measure for 100 Monte-Carlo iterations for SVM-POLY classifier.

*p*	θ	Precision (%)	Recall (%)	Accuracy (%)	f-Measure (%)
2	30	75.24	88.92	78.53	81.51
2	36	77.82	85.91	79.24	81.67
2	42	77.54	86.27	79.32	81.67
2	48	78.35	86.98	79.54	82.44
2	56	78.78	86.11	79.23	82.28
3	30	75.39	87.92	79.21	81.17
3	36	76.41	87.69	79.11	81.66
3	42	76.12	87.77	78.92	81.53
3	48	78.01	87.34	78.24	82.41
3	56	78.09	86.92	77.88	82.27
4	30	77.98	85.43	77.87	81.54
4	36	77.15	86.13	77.46	81.39
4	42	77.26	86.24	77.79	81.50
4	48	78.65	86.71	77.92	82.48
4	56	78.92	87.01	78.01	82.77

**TABLE 4:** Averages of precision, recall, accuracy, and f-measure for 100 Monte-Carlo iterations for SVM-RBF classifier.

C	σ	Precision (%)	Recall (%)	Accuracy (%)	f-Measure (%)
1,000	30	78.66	83.41	79.58	80.97
1,000	36	77.65	83.45	79.61	80.45
1,000	42	78.24	83.98	79.54	81.01
1,000	48	78.54	84.14	80.13	81.24
1,000	56	78.98	84.35	79.98	81.58
3,000	30	79.13	83.85	79.32	81.42
3,000	36	79.24	83.24	79.45	81.19
3,000	42	79.47	84.25	79.33	81.79
3,000	48	80.13	84.27	80.24	82.15
3,000	56	79.91	82.78	80.76	81.32
5,000	30	73.37	89.86	80.18	80.78
5,000	36	74.27	88.24	80.27	80.65
5,000	42	73.45	88.15	80.13	80.13
5,000	48	73.26	88.97	80.54	80.35
5,000	56	73.15	88.48	79.93	80.09

**TABLE 5:** Averages of precision, recall, accuracy, and f-measure for 100 Monte-Carlo iterations for BPN classifier.

Neurons	Precision (%)	Recall (%)	Accuracy (%)	f-Measure (%)
100	72.28	90.21	80.52	80.26
120	72.37	89.25	80.24	79.93
140	72.56	89.76	80.52	80.25
160	72.89	89.91	80.59	80.51
180	73.24	90.73	81.11	81.05

**TABLE 6:** Averages of precision, recall, accuracy, and f-measure for 100 Monte-Carlo iterations for random forest classifier.

Decision trees	Precision (%)	Recall (%)	Accuracy (%)	f-Measure (%)
20	92.24	93.47	91.24	92.85
40	92.37	94.13	91.78	93.24
60	93.91	93.71	91.25	93.81
80	93.63	94.61	91.51	94.12
100	93.24	94.28	91.39	93.76

#### 4.2.2 Random Forest Boosted Version

In order to have a more clear view about the potential of random forest, we simulate different schemas of estimators and calculate again precision, recall, accuracy, and f-measure. In this simulation scenario, denoted hereafter as RF-Boost, five weak learners (or five random forest classifiers) were used where the estimator of each one of the weak learners is estimators = [40, 60, 80, 100, 120] decision trees. The simulation results of RF-Boost scenario are given in Table 7. Comparing Tables 6, 7, one can see that, with the RF-Boost scenario, the predictive performance is increased by 3–4% on f-measure, a fact that indicates the dominance of boosted form compared to any other predictive approach, tested here.

**TABLE 7:** Averages of precision, recall, accuracy, and f-measure for 100 Monte-Carlo iterations of AdaBoost on random forest classifier.

Parameters of weak learners	Precision (%)	Recall (%)	Accuracy (%)	f-Measure (%)
20, 40, 60, 80, 100	97.63	97.28	95.32	97.45

#### 4.2.3 Unsupervised Learning Results

As mentioned before, two of the available injection molding machines lack ground truth values. The simulation results of the unsupervised training applied to these machines are shown in Table 8. The values of the table refer to both machines trained with historical data. According to the evaluation metric description in Section 4.1 it seems that One-Class SVM is the weaker learner and LOF and DBSCAN have given better results in machines five and six, respectively. The challenge of the unlabeled data is to be well evaluated in order to use the corresponding models as fault detectors. It may be a weaker method compared to supervised evaluation but the system aims to give a handful solution in case where ground truth is missing and give accurate results in cooperation with the ensemble enhancement given by majority voting and also with the retraining module of the proposed methodology.

**TABLE 8:** Unsupervised evaluation metrics on injection molding machine 5/6.

Algorithms	Injection machine	Silhouette	Davies–Bouldin	Calinski–Harabasz	Dunn
LOF	Machine 5	0.05	4.57	119.89	0.11
	Machine 6	0.11	2.83	400.44	0.03
K-means	Machine 5	0.15	3.29	774.63	0.05
	Machine 6	0.21	2.16	1,041.22	0.02
DBSCAN	Machine 5	0.05	4.80	1,456.17	0.02
	Machine 6	0.42	1.51	1,449.55	0.41
One-Class SVM	Machine 5	0.05	17.18	134.61	0.01
	Machine 6	0.01	18.52	98.59	0.01

### 4.3 Cognitive Mechanism Testing

The prediction models generated by the online training (Section 3.2) are used in live prediction phase (Section 3.4) where real-time data are monitored and anomalies are detected. The cognitive mechanism operates at the same time as live prediction and triggers the retraining of a prediction model when needed. In order to test this feature, the performance of models was recorded as live prediction and cognitive check are operating. Specifically, four prediction models are monitored: decision tree and random forest models for the supervised learning of labeled data and DBSCAN and K-means models for the unsupervised learning of unlabeled data. The accuracy evaluation metric is recorded for the supervised learning and the silhouette score for the unsupervised. The model performance was recorded for as long as it takes for the cognitive mechanism to trigger 200 retraining times of the models.

**FIGURE 3:**
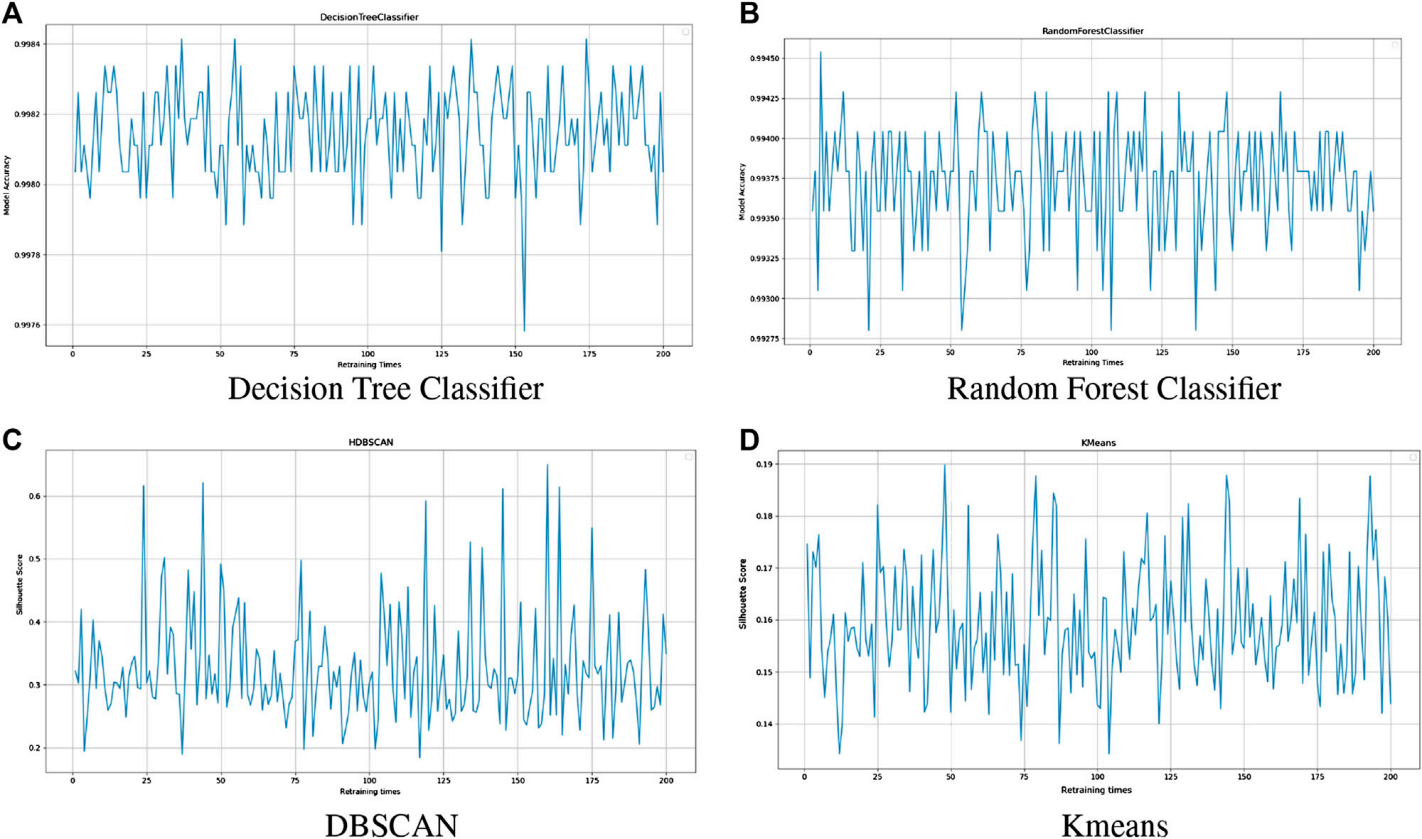
Cognitive mechanism performance monitoring. The horizontal axis of the graphs shows the times of retraining and the vertical axis shows the accuracy **(A,B)** and silhouette score **(C,D)** of the model.

Figure 3 shows some indicative results of this testing. It illustrates the diagrams of the evaluation metric throughout 200 retraining times of a single model (accuracy for supervised and silhouette score for unsupervised learning). The diagrams indicate that the models’ performance is maintained in high levels after the model retraining: decision tree classifier’s accuracy does not fall under 0.997 and random forest under 0.992 and DBSCAN and K-means’ silhouette scores are kept over 0.2 and 0.14, respectively. The model retraining is triggered by variations noticed in the real-time data compared with the historical ones, so it is crucial that it will be accomplished the time that is being triggered, regardless of the results that it will induce.

As a follow-up to the above diagrams, the times where the model was improved after retraining were calculated. Table 9 shows that most of the times the execution of retraining improves the performance of the model. The aim of the retraining module is to automatically update the predictive models when their performance diverges. These indicative results explain the need of the complete system to be updated occasionally in order to be able to maintain high quality and accuracy in the anomaly detection pipeline.

**TABLE 9:** The percentage of retraining times that accuracy improvement is noticed.

Algorithm	Machine	Retraining improvement (%)
Decision tree classifier	Injection molding machine 1	63
Random forest	Injection molding machine 2	63
K-means	Injection molding machine 5	75
DBSCAN	Injection molding machine 6	73

### 4.4 Comparison with Prior Work

In order to support our proposed methodology, we present a determinate comparison between our methodology and other works from literature. The comparison concerns the studies that deal with injection molding machines as this is the core of our research and focuses on aspects of each proposed methodology, since the results of each work are disparate or unavailable. The first study includes an injection molding machine’s use case and explores the available data while the machine operates (Gatica et al., 2016). The study extracts normal behavior models and notices deviations from the expected behavior. The authors use trend analysis functions to predict already known failures and achieve reduction of machine downtime.

The second study deals with anomaly detection on streaming data applied to injection molding machines (Jankov et al., 2017). A sliding window observes the streaming data and finds clusters by using K-means algorithm. The clusters are used for training of a Markov model for the window. New models are trained as the window slides over new data. Anomalies are detected in the streaming data by calculating transition probability and comparing it with a threshold. The advantage of Jankov et al. (2017) work is the computational capabilities of the system which is augmented by real-time parallel task distribution.

Park et al. (2016) address the problem of machine condition monitoring by identifying the injection molding operational parameters. Statistical analysis is applied to these parameters in order to distinct the most significant ones. Real-time data series are monitored by prediction models and the results are evaluated by Nelson rules. The method detects abnormal patterns of the parameters and identifies the machine parts where maintenance actions should aim.

Our proposed method follows a similar approach to the aforementioned works. The aim is to investigate abnormal operations in the injection molding process starting with data analysis and resulting in prediction models that determine anomalies. In contrast with the studies above, we proposed a methodology which can handle both labeled and unlabeled data and also address the challenge of unknown errors in case of machine abnormal behavior. Additionally, the other works derive prediction models using one specific analytical method, but the current study includes multiple classification and clustering learners for the generation of prediction models. From the set of trained models, the one with higher performance will be used for real-time anomaly monitoring. Also, there is the capability of meta-learning as described in Section 3.3.

The distinguishing feature of the current work though is the constant updating of the system’s prediction models through cognitive retraining. Except Jankov et al. (2017) work which trains new prediction models as they cross by data streams, the remaining studies do not focus on the potential changes that can be noticed in live data or the possible degradation of the prediction methods. This is the core of the cognition aspect of the current work which achieves steady performance of the real-time anomaly detection system.

## 5 Conclusions

In this paper a cognitive analytics application is presented, focusing on predictive maintenance applied to injection molding machines. A complete solution was described in detail including different stages of training of historical data, live prediction on real-time data, and automated retraining which aims to keep the prediction process up to date. The proposed solution manages both labeled and unlabeled datasets and applies ensembles methods to top of individual supervised and unsupervised learners. The generated prediction models receive real-time data streams and perform anomaly detection on the features of the injection molding machine measurements. A cognitive mechanism was developed and tested, which monitors the dataset changes, on the one hand, and the model performance, on the other hand, and constantly updates the predictive models.

The main findings of our research are summarized below:

- The proposed solution achieves combining different training methods and detecting faults in different machines, located in the same factory site.
- Ensemble methods can enhance the prediction models’ performance results.
- Automatic updating of trained models addresses the problem of possible deviations of new incoming machine data or potential prediction models’ degradation.
- High model performance is preserved in real-time anomaly detection and data monitoring through automatic triggering of model retraining.

As a result of these assets, the presented method can constitute an assisting tool for the decision support system of factory sites facilitating injection molding machines, in order to prevail failures in production and downtime of machines.

Current ongoing work is implementing the creation of user interfaces for the proposed real-time anomaly detection methodology. Advanced visualizations are incorporated, offering an enhanced user experience and a thorough view of raw data, processed and clean data, model training, evaluation, and results, as well as real-time monitoring. Two user views are set up: the data scientist view and the regular end user. The data scientist can choose parameters and methods for online training which will operate the live monitoring for the regular end user. The anomalies are detected and visualized so as the predictive maintenance manager can make the necessary decisions in case of machine abnormalities.

Future work will concentrate on applying the presented methodology to different machine data of the Industry 4.0 domain and investigate a generic cognitive analytics framework for predictive maintenance. The development of more learning techniques is being considered as a next step, especially regarding the field of ensembled methods. Lastly, there is definitely a room for improvement in the unsupervised learning area regarding evaluation and meta-learning processes.

## Data Availability Statement

The data analyzed in this study are subject to the following licenses/restrictions: The dataset is private and anonymized. Requests to access these datasets should be directed to vrousop@iti.gr.

## Author Contributions

This paper is a joint work from all listed authors. In particular, VR and AN contributed to conceptualization and writing of the first draft of the document. VR is also the main contributor in presented framework’s development. Furthermore, TV wrote the section related to ensemble methods and contributed to framework’s design alongside VR and AN. DI and DT supervised the work and provided the necessary design guidelines. All the authors contributed to changes and corrections before the final submission.

## Conflict of Interest

The authors declare that the research was conducted in the absence of any commercial or financial relationships that could be construed as a potential conflict of interest.
